# Phenocoll Hydrochlorate

**Published:** 1892-07-23

**Authors:** 


### THERAPEUTICS.

### PHENOCOLL HYDROCHLORATE.

This substance, the nature and properties of which we described recently in these pages, has been used for rheumatism, malaria, and as an antipyretic. It shares with ordinary phenacetin a reputed exemption from injurious after-effects. Ott, indeed, was able by injection into frogs of two-grain doses to bring the heart to a standstill in diastole, and in rabbits a fall in blood pressure and in the pulse rate followed injection of large doses; but Kobert and Von Mering claim that it is practically harmless to animals, and no ill effects in human beings appear to have been discovered. One of its most recent applications has been in the treatment of malarial fevers, where it is claimed to have a therapeutic action exceeding in some cases that of quinine. Albertoni states that he was not merely able to cut short the attacks by its use, but even to effect a permanent cure in twenty-four out of thirty-four cases in which he employed it. He gave fifteen-grain doses several hours before the attack and continued the administration once a day for a short time. It appears to have some power in relieving the pains of rheumatism, apart from its action on the temperature; and from Ott's experiments, this action is probably due to its direct influence on the spinal cord. According to Dr. Hertel, it succeeded in some of those troublesome cases of acute rheumatism where salicylates have no effect, so far, at least, as the relief of the pains was concerned. A remedy of use in these obstinate cases is not to be despised, for at present we have no means of diagnosing that variety of acute rheumatism which is acted on by salicylates from those where it is inoperative. Possibly, these instances may depend on some failure of elimination, for at times the addition of a diuretic, such as scoaprium, to the salicylates will effect improvement. Fifteen-grain doses of phenocoll may here be given every five or six hours for 24 or 30 hours with benefit. The same amount as a maximum is employed for reducing high temperatures. The anti-pyrectic power appears to be slightly greater than that of phenacetin, and twice as great as that of antipyrin. No collapse or sweats are said to follow its use, though the urine at times becomes dark brown. Like phenacetin, phenocoll is not very soluble, but the hydro-chlorate in water slightly above the ordinary temperature dissolves easily, and the taste can be masked by a little syrup. If further experience should bear out its claims, we shall have a useful alternative to the older remedies for hyperexia, rheumatic and neuralgic affections

